# Association between fasting plasma glucose and nonalcoholic fatty liver disease in a nonobese Chinese population with normal blood lipid levels: a prospective cohort study

**DOI:** 10.1186/s12944-020-01326-3

**Published:** 2020-06-20

**Authors:** Yang Zou, Meng Yu, Guotai Sheng

**Affiliations:** 1 Department of Cardiology, Jiangxi Provincial People’s Hospital, No. 92 Aiguo Road, Donghu District, Nanchang, 330006 Jiangxi Province China; 2 Department of Graduate School, Medical College of Nanchang University, No. 461 Bayi Avenue, Donghu District, Nanchang, 330006 Jiangxi Province China

**Keywords:** Nonobese, Nonalcoholic fatty liver disease, Fasting plasma glucose, Chinese, Normal blood lipid levels, Prospective cohort study

## Abstract

**Background:**

Fasting plasma glucose (FPG) is an easily quantifiable and inexpensive metabolic marker, which is often used to assess cardiovascular disease and diabetes. However, there have been limited studies on the association between FPG and nonalcoholic fatty liver disease (NAFLD) risk in nonobese people, especially in Chinese individuals. The purpose of this study was to investigate the association between FPG and NAFLD in nonobese Chinese people with normal blood lipid levels.

**Methods:**

In this prospective cohort study, 9767 nonobese participants with normal blood lipid levels without NAFLD were recruited and prospectively followed for 5 years. The Cox proportional hazard model was used to evaluate the risk factors of NAFLD. Moreover, a Cox model with cubic spline functions and smooth curve fitting (the cubic spline smoothing) were used to identify the nonlinear association between FPG and NAFLD.

**Results:**

During the 5-year follow-up, 841 (8.61%) participants were diagnosed with NAFLD. The good functional results (without NAFLD) estimated by the Kaplan-Meier method for 1 year, 2 years, 3 years, 4 years, and 5 years were 98.84, 95.35, 91.67%, 87.57 and 74.86%, respectively. Additionally, through the Cox proportional hazard model, after adjusting for other covariates, there was an independent positive correlation between FPG and increased NAFLD risk (HR:1.21, 95% CI:1.15–1.28, *P* < 0.0001), and the NAFLD risk was incrementally higher with the rising FPG quartile. The nonlinear association between FPG and NAFLD was visualized by cubic spline smoothing technique. It was calculated that the inflection point of FPG was 5.54. When FPG ≤ 5.54, there was a positive correlation between FPG and the risk of NAFLD (HR:2.20, 95% CI:1.78–2.73, *P* < 0.0001). When FPG > 5.54, the risk of NAFLD increased by 50% (HR:1.10, 95% CI:1.02–1.18, *P* = 0.0159) compared with the left side of the inflection point and gradually leveled off.

**Conclusions:**

In a nonobese Chinese population with normal lipid levels, there is an independent nonlinear association between FPG and NAFLD, and the increase in FPG may indicate an increased risk of NAFLD. Additionally, this independent association is more obvious in the short stature population.

## Background

NAFLD is a collection of liver diseases such as hepatic steatosis, nonalcoholic steatohepatitis (NASH) and hepatic fibrosis and is closely related to cardiovascular disease, chronic kidney disease, and type 2 diabetes [1–3]. The effect of NAFLD is not limited to the liver. It has varying degrees of adverse effects on many organs/systems of the whole body. Some studies have highlighted that NAFLD should be considered not only as a specific disease of the liver but also as an early vector of multisystem diseases [2–5]. Younossi ZM and his team systematically analyzed the global epidemiology of NAFLD, which showed a troubling result; at present, the estimated global combined prevalence rate of NAFLD is 27.4% (95% CI:23.3–31.9%), which means that up to a quarter of people around the world are being influenced by NAFLD. The authors also pointed out that the incidence of NAFLD has further increased with the prevalence of obesity [6]. Previous studies have also indicated that NAFLD is more common in obese people [2, 7, 8]. However, there are still many nonobese people diagnosed with NAFLD in clinical work, especially in Asia [9–11]. Epidemiological surveys showed that the prevalence of NAFLD in Asia was approximately 25%, of which approximately 8% of NAFLD patients are nonobese Asians [10]. Dyslipidemia is a pivotal contributor to NAFLD [12, 13]. Currently, published work on the risk of NAFLD in nonobese people with normal blood lipid levels is limited [14, 15]. This substantial particular population has great potential research value.

FPG has been proven to be an important risk factor for diabetes and cardiovascular disease in the past, and it is often used to evaluate the condition and prognosis [16, 17]. Recently, some clinical studies from Sri Lanka and Taiwan have reported an association between FPG and the development of NAFLD. The reports argued that higher FPG is a significant risk factor for NAFLD [18, 19]. However, the sample size of these studies was relatively small, and similar studies are still lacking. Given the prevalence of NAFLD, its potential economic severity and disease burden, and the immature data on the incidence of NAFLD in nonobese people [20], the present study was designed to clarify the correlation between FPG and NAFLD among nonobese Chinese with normal blood lipid levels.

## Methods

### Study population and design

The clinical data of the study population came from a public database (https://datadryad.org, 10.5061/dryad.1n6c4), which was provided by Sun et al. [15]. The study design used here has been described in a previous study [15]. Briefly, this was a prospective cohort study of 16,173 participants recruited at Wenzhou Medical Center of Wenzhou People’s Hospital between January 2010 and December 2014. The exclusion criteria in this study were as follows: (1) body mass index (BMI) ≥25 kg/m^2^; (2) self-reported excessive drinking> 140 g/week for men and > 70 g/week for women; (3) patients with chronic liver disease; (4) accompanied with dyslipidemia (LDL-C > 3.12 mmol/L, triglyceride (TG) > 1.7 mmol/L, total cholesterol (TC) > 5.2 mmol/L, high-density lipoprotein cholesterol (HDL-C) < 1.04 mmol/L); and (5) were taking antihypertensive, hypoglycemic, or lipid-lowering drugs. In this study, the participants’ personal information was processed anonymously and replaced by a health examination number. The research ethics approval was obtained in the previous study and was no longer required for the present study.

### Data collection

A standardized self-filling spreadsheet designed by specially trained medical personnel was used to collect general clinical baseline information, including age, sex, weight, height, diastolic blood pressure (DBP) and systolic blood pressure (SBP). Other hematologic indexes including BUN (blood urea nitrogen), FPG, UA (uric acid), Cr (creatinine), TC, TG, HDL-C, LDL-C, ALT (alanine aminotransferase), AST (aspartate aminotransferase), GGT (gamma-glutamyl transferase), ALP (Alkaline phosphatase), ALB (albumin) TP (Total Protein), DBIL (Direct bilirubin), GLB (globulin) and TB (Total bilirubin) were measured on an autoanalyzer (Abbott AxSYM).

### Diagnosis of NAFLD

Diagnosis of NAFLD followed the “*Chinese Guideline on Diagnosis and Treatment of NAFLD”* [21]. The diagnostic criteria should meet two of the following five abnormalities echoes in the abdominal color Doppler ultrasound examination, the first of which was necessary for diagnosis: (1) diffuse hyperechoic liver relative to spleen and kidney; (2) reduced visibility of detailed structure in the liver; (3) mild to moderately enlarged liver with blunt, rounded edges; (4) weakened hepatic blood flow signal with normal blood flow distribution; and (5) unclear or nonintact display of envelope of right liver lobe and diaphragm.

### Follow-up

The start time of the follow-up was considered to be that after the clinicians collected complete data and assessed the condition of NAFLD. All study participants were contacted for follow-up once a year using the same procedure as baseline information collection. Hematological indices and liver ultrasound tests were performed in the same laboratory as before to determine the incidence of NAFLD. All patients were prospectively followed for 5 years. The endpoint of follow-up was incident NAFLD.

### Statistical analysis

Statistical analyses were performed using the software R (version 3.4.3) and Empower (R) (www.empowerstats.com). To better understand the association between FPG and NAFLD, the study population was grouped based on FPG quartiles (Q1: < 4.72, Q2: > 4.72, < 4.97, Q3: > 4.97, < 5.27, Q4: > 5.27). Continuous variables were summarized as the mean ± standard deviation. The Kolmogorov-Smirnov test was used to measure the normal distribution of values since all continuous variables were non-normally distributed and so the differences between each group compared by the Kruskal-Wallis H test. Categorical variables were described as n or %, and differences between groups were compared using χ^2^-test. The collinearity between variables was tested using multiple linear regression based on the variance inflation factor (VIF) [22]. Variables with VIF > 5 were considered to have severe multicollinearity. The Kaplan-Meier method was also performed to calculate the cumulative NAFLD incidence rate function of events over time, and the survival curve functions between FPG quartile were compared using the log-rank test. A univariable and multivariable Cox proportional hazards model was developed to determine the independent risk factors of NAFLD events. First, all variables were evaluated by univariate analysis, then significant variables in the univariate analyses (*P* < 0.05) or those considered to be of clinical significance were incorporated into the multivariate analysis. Subsequently, stepwise multivariable regression analysis was performed. Additionally, the Cox proportional hazard regression model was used to evaluate each FPG quartile to the risk of NAFLD. Meanwhile, the results of the unadjusted analysis (crude model), minimum adjustment analysis (model I adjust for: sex, age, and BMI), and full adjustment analysis (model II adjusts for sex, age, Cr, UA, TC, TG, HDL-C, LDL-C, height, BMI, ALP, GGT, ALT, AST, ALB, GLB, DBIL, SBP, and DBP) were presented based on the Strengthening the Reporting of Observational Studies in Epidemiology (STROBE) statement [23]. The hazard ratios (HR) with 95% confidence intervals (CI) were recorded. Moreover, a Cox proportional hazards regression with cubic spline functions and smooth curve fitting (the cubic spline smoothing) were used to address the nonlinear association between FPG and NAFLD. If a nonlinear association was observed, a two-piecewise linear regression model was used to calculate the threshold effect and look for the inflection point of two straight lines (recursive method). To avoid the deviation caused by the difference of the FPG level, a sensitivity analysis was used to verify the reliability of the conclusion further by transforming FPG into a categorical variable and calculating the trend *P.* Additionally, the subgroup analyses were performed using Cox proportional hazard model, and the interactions of subgroups were tested by likelihood ratio tests (adjustment was made for clinically significance variables and univariate analysis variables with *P* < 0.05). To maximize the statistical efficiency and reduce the bias that may be caused by exclusion due to the absence of some variables, the mean or median was used to fill in missing data for continuous variables (ALP (*n* = 2543), GGT (*n* = 2545), AST (*n* = 2543), ALT (*n* = 2543), TP (*n* = 871), ALB (*n* = 871), GLB (*n* = 871), TB (*n* = 3466), DBIL (*n* = 4378), SBP (*n* = 8), DBP (*n* = 8)). All analyses in this paper were carried out using complete data, and the significance standard was considered to be bilateral at *P* < 0.05.

## Results

### Description of the study groups

A total of 16,173 participants were recruited for this study. After screening according to the exclusion criteria, 9767 participants were finally enrolled in the cohort study, including 5022 males (51.42%) and 4745 females (48.58%), and the average age was 42.46 ± 14.71 years. The baseline characteristics of the study population after the quartile grouping of FPG are summarized in Table 1; it was observed that there were significant differences among all groups except TB. Compared with the lower FPG group (≤4.97), participants in the higher FPG group (> 4.97) were generally older and had higher height, weight, BMI, BUN, Cr, UA, FPG, TC, TG, LDL-C, ALP, GGT, ALT, TP, GLB, SBP, and DBP. Moreover, the incidence of NAFLD increases gradually with the increase of FPG level (Q1:4.21% vs. Q2:6.55% vs. Q3:8.50% vs. Q4:15.03%), on the contrary, HDL-C gradually decreased with the increase of FPG quartile.

**Table 1:** Baseline Characteristics of participants (*N* = 9767)

Variables	FPG Quartiles				*P*-value
	Q1(≤4.72)	Q2(> 4.72, ≤4.97)	Q3(> 4.97, ≤5.27)	Q4(> 5.27)	
Age, years	41.40 ± 14.08	41.18 ± 14.02	42.31 ± 14.76	44.90 ± 15.62	< 0.001
Sex					< 0.001
Women	1237 (51.05%)	1197 (50.23%)	1177 (47.21%)	1134 (45.95%)	
Men	1186 (48.95%)	1186 (49.77%)	1316 (52.79%)	1334 (54.05%)	
NAFLD	102 (4.21%)	156 (6.55%)	212 (8.50%)	371 (15.03%)	< 0.001
Weight, kg	54.84 ± 7.89	56.21 ± 8.07	57.35 ± 8.48	58.81 ± 8.19	< 0.001
Height, m	1.63 ± 0.07	1.64 ± 0.08	1.65 ± 0.08	1.65 ± 0.08	< 0.001
BMI, kg/m^2^	20.54 ± 2.00	20.86 ± 2.02	21.10 ± 2.03	21.59 ± 1.99	< 0.001
BUN, mmol/L	4.29 ± 1.27	4.30 ± 1.16	4.45 ± 1.28	4.81 ± 1.54	< 0.001
Cr, mmol/L	72.13 ± 21.69	74.85 ± 16.79	77.32 ± 19.84	83.66 ± 36.67	< 0.001
UA, μmol/L	255.92 ± 75.13	257.89 ± 80.45	262.07 ± 77.78	278.24 ± 83.90	< 0.001
FPG, mmol/L	4.50 ± 0.19	4.84 ± 0.07	5.11 ± 0.09	5.82 ± 0.97	< 0.001
TC, mmol/L	4.29 ± 0.54	4.33 ± 0.53	4.35 ± 0.52	4.39 ± 0.53	< 0.001
TG, mmol/L	0.90 ± 0.30	0.95 ± 0.31	0.97 ± 0.30	1.05 ± 0.31	< 0.001
HDL-C, mmol/L	1.54 ± 0.31	1.53 ± 0.30	1.51 ± 0.29	1.49 ± 0.29	< 0.001
LDL-C, mmol/L	2.05 ± 0.41	2.10 ± 0.41	2.13 ± 0.41	2.17 ± 0.42	< 0.001
ALP, U/L	67.34 ± 15.56	67.80 ± 18.67	68.52 ± 18.42	73.06 ± 23.48	< 0.001
GGT, U/L	20.77 ± 12.31	21.69 ± 14.20	22.91 ± 17.48	28.48 ± 29.65	< 0.001
ALT, U/L	16.97 ± 13.67	17.16 ± 10.43	17.28 ± 16.08	19.15 ± 14.23	< 0.001
AST, U/L	21.62 ± 7.94	21.42 ± 6.93	21.53 ± 6.91	23.04 ± 9.06	< 0.001
TP, g/L	73.30 ± 3.82	73.65 ± 3.73	73.88 ± 3.85	74.11 ± 4.17	< 0.001
ALB, g/L	44.15 ± 2.66	44.38 ± 2.45	44.32 ± 2.63	44.31 ± 2.70	0.044
GLB, g/L	29.15 ± 3.62	29.26 ± 3.46	29.55 ± 3.56	29.80 ± 4.06	< 0.001
TB, μmol/L	11.88 ± 3.92	11.74 ± 3.84	11.76 ± 3.99	11.90 ± 4.14	0.304
DBIL, μmol/L	2.29 ± 0.92	2.19 ± 0.83	2.21 ± 0.86	2.26 ± 0.95	< 0.001
SBP, mmHg	112.85 ± 13.34	115.01 ± 14.22	118.06 ± 15.29	126.13 ± 17.82	< 0.001
DBP, mmHg	68.62 ± 9.01	70.14 ± 9.50	71.67 ± 9.90	74.65 ± 10.32	< 0.001

Values are n(%) or mean ± SD

*Abbreviations*: *BMI* Body mass index, *NAFLD* Nonalcoholic fatty liver disease, *BUN* Blood urea nitrogen, *Cr* Creatinine, *UA* Uric acid, *FPG* Fasting plasma glucose, *TC* Total cholesterol, *TG* Triglyceride, *HDL-C* High-density lipoprotein cholesterol, *LDL-C* Low-density lipoprotein cholesterol, *ALP* Alkaline phosphatase, *GGT* Gamma-glutamyl transferase, *ALT* Alanine aminotransferase, *AST* Aspartate aminotransferase, *TP* Total Protein, *ALB* Albumin, *GLB* Globulin, *TB* Total bilirubin, *DBIL* Direct bilirubin, *DBP* Diastolic blood pressure, *SBP* Systolic blood pressure

### Follow-up results

**Fig. 1:**
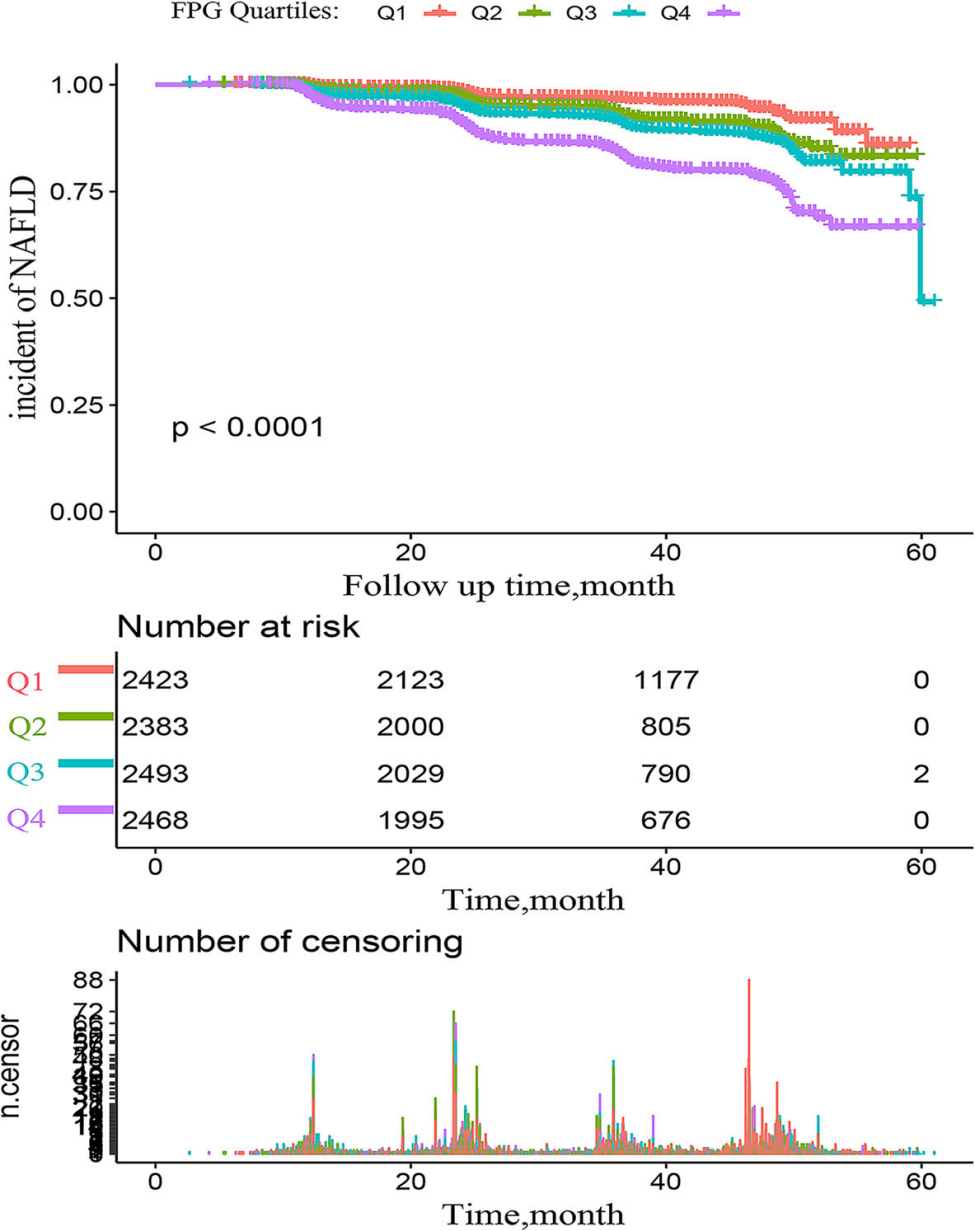
Kaplan–Meier analysis of incidence of NAFLD based on FPG quartiles (*P* < 0.0001)

During the five-year follow-up, 841 (8.61%) nonobese participants were diagnosed with NAFLD. Kaplan-Meier estimated that the good functional results (without NAFLD) in 1 year, 2 years, 3 years, 4 years and 5 years were 98.84% (98.62, 99.05%), 95.35% (94.9, 95.79%), 91.67% (91.03, 92.31%), 87.57% (86.68, 88.47%) and 74.86% (67.15,83.64%), respectively. Figure 1 shows the Kaplan-Meier curve of NAFLD event risk according to quartiles of FPG. It could be observed that there was a significant difference in the risk of NAFLD between FPG groups (log-rank test *P* < 0.0001); with increasing FPG, the cumulative risk of NAFLD gradually increases.

### Association of baseline variables with NAFLD risk

Through univariate and multivariate analysis (adjusted for sex, age, Cr, UA, FPG, TC, TG, HDL-C, LDL-C, height, BMI, ALP, GGT, ALT, AST, ALB, GLB, DBIL, SBP, and DBP), the variables associated with the risk of NAFLD were determined (Table 2). The results showed that even in people with normal blood lipid levels and nonobese population, height (HR:2.95, 95% CI:1.07–8.16, *P* = 0.037), BMI (HR:1.69, 95% CI:1.62–1.78, *P* < 0.0001), TG (HR:2.73, 95% CI:2.12–3.50, *P* < 0.0001), LDL-C (HR:2.53, 95% CI:1.80–3.56, *P* < 0.0001) and FPG (HR:1.21, 95% CI:1.15–1.28, *P* < 0.0001) were still strong independent risk factors for NAFLD.

**Table 2:** Cox proportional hazard associations of NAFLD risk in the study population

Variables	Univariable		Multivariable	
	HR (95%CI) *P*-value	VIF	HR (95%CI)	*P*-value
Sex (men)	1.13 (0.98, 1.29) 0.0845	1.1	0.89(0.78, 1.03)	0.1301
Age	1.01 (1.00, 1.01) 0.0005	1.1	1.01(1.00, 1.01)	0.0077
Weight	1.11 (1.10, 1.12) < 0.0001	> 5		
Height	82.07(34.65,194.36) < 0.0001	1.4	2.95(1.07, 8.16)	0.0370
BMI	1.93 (1.84, 2.02) < 0.0001	1.3	1.69(1.62, 1.78)	< 0.0001
BUN	1.00 (0.95, 1.05) 0.9984	1.4		
Cr	1.00 (1.00, 1.01) < 0.0001	1.6	0.99(0.99, 1.00)	0.5999
UA	1.00 (1.00, 1.01) < 0.0001	1.6	1.00(0.99, 1.00)	0.3454
FPG	1.32 (1.28, 1.37) < 0.0001	1.2	1.21(1.15, 1.28)	< 0.0001
TC	1.40 (1.22, 1.60) < 0.0001	4.7	0.58(0.44, 0.76)	< 0.0001
TG	8.22 (6.65, 10.15) < 0.0001	1.4	2.73(2.12,3.50)	< 0.0001
HDL-C	0.25 (0.19, 0.33) < 0.0001	2	0.88(0.63, 1.25)	0.4858
LDL-C	3.24 (2.70, 3.88) < 0.0001	4.5	2.53(1.80, 3.56)	< 0.0001
ALP	1.01 (1.01, 1.02) < 0.0001	1.2	1.004(1.00, 1.007)	0.0157
GGT	1.01 (1.01, 1.02) < 0.0001	1.3	1.005(1.004, 1.007)	< 0.0001
ALT	1.01 (1.01, 1.02) < 0.0001	3.1	1.02(1.01, 1.02)	< 0.0001
AST	1.02 (1.01, 1.02) < 0.0001	3.2	0.98(0.96, 1.01)	0.0011
TP	1.01 (0.99, 1.02) 0.5606	> 5		
ALB	0.97 (0.95, 1.00) 0.0389	1.2	0.99(0.96, 1.01)	0.2893
GLB	1.02 (1.00, 1.04) 0.0358	1.1	0.99(0.97, 1.01)	0.5221
TB	1.01 (0.99, 1.03) 0.3324	1.8		
DBIL	0.51 (0.45, 0.59) < 0.0001	1.9	0.52(0.45, 0.59)	< 0.0001
SBP	1.03 (1.02, 1.03) < 0.0001	2.5	0.99(0.97, 1.00)	0.1210
DBP	1.05 (1.04, 1.05) < 0.0001	2.2	1.02(1.01, 1.03)	< 0.0001

*Abbreviations*: *CI* Confidence, *HR* Hazard ratios; other abbreviations as in Table 1

### Association between FPG and NAFLD in the nonobese population

**Fig. 2:**
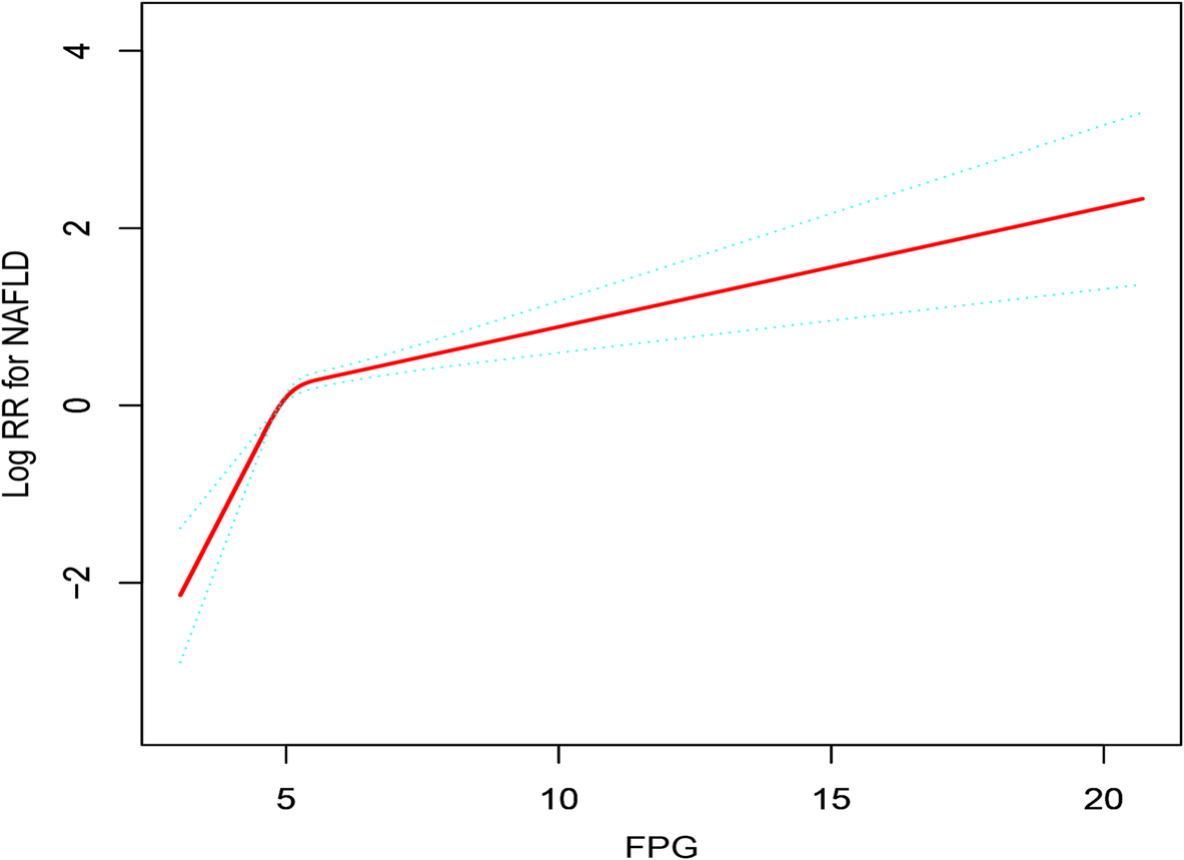
The nonlinear relationship between FPG and the incidence of NAFLD (adjusted for Sex, Age, Cr, UA, TC, TG, HDL-C, LDL-C, Height, BMI, ALP, GGT, ALT, AST, ALB, GLB, DBIL, SBP, and DBP)

Before the establishment of the Cox proportional hazard model, the collinearity between variables was screened. Variables with VIF > 5 were considered as showing severe multicollinearity and cannot be included in the multivariate regression equation. For details of collinear screening, see Supplementary Table 1. Table 3 summarizes the association between FPG and NAFLD in nonobese people. It can be seen that the core results of the three models were consistent, and there was a positive correlation between FPG and the risk of NAFLD. After adjusting the full model (model II), the risk of NAFLD increases by 1.21 times per 1 mmol increase of FPG (HR:1.21, 95% CI:1.15–1.28, *P* < 0.0001). It was worth noting that there was a nonlinear association between FPG and the risk of NAFLD was visualized by cubic spline smoothing technique, and this association still exists after adjusting for other covariables (Fig. 2). The inflection point of FPG was calculated to be 5.54 (Table 4), and there was a significant difference between the left and right sides of the inflection point (Log-likelihood ratio test *P* < 0.001). When FPG ≤ 5.54, the risk of NAFLD increased by 2.2 times per 1 mmol/l increase in FPG. Similarly, the positive association can be seen between FPG and NAFLD on the right side of the inflection point (FPG > 5.54), as the risk of NAFLD increased by 50% compared to the left (HR:1.10, 95% CI:1.02–1.18, *P* = 0.0159). Additionally, sensitivity analysis showed that, after review of all NAFLD events, the change of the effect value of FPG quartile (1,1.29,1.41,2.25) indicated an increase in FPG content, and the risk trend of NAFLD gradually increased (*P* for trend< 0.00001). After the same procedures were carried out in the original data (including missing data), the core results of the analysis of the original data and the complete data were consistent, further supporting the reliability of the results of this study (Supplementary Table 2, Supplementary Table 3 and Supplementary Figure 1).

**Table 3:** Relationship between FPG and NAFLD in different models

Variable	Crude Model	Model I	Model II
	HR (95%CI) *P*-value	HR (95%CI) *P*-value	HR (95%CI) *P*-value
FPG	1.32 (1.28, 1.37) < 0.0001	1.25 (1.19, 1.30) < 0.0001	1.21 (1.15, 1.28) < 0.0001
FPG (quartile)			
Q1	Ref	Ref	Ref
Q2	1.80 (1.40, 2.31) < 0.0001	1.46 (1.14, 1.87) 0.0031	1.29 (1.01, 1.66) 0.0450
Q3	2.35 (1.86, 2.98) < 0.0001	1.72 (1.35, 2.18) < 0.0001	1.41 (1.11, 1.79) 0.0054
Q4	4.45 (3.57, 5.54) < 0.0001	2.70 (2.17, 3.38) < 0.0001	2.25 (1.79, 2.82) < 0.0001
*P* for trend	< 0.0001	< 0.0001	< 0.0001

The crude model adjusts for: None Model I adjust for: sex, age, and BMI. Model II adjusts for sex, age, Cr, UA, TC, TG, HDL-C, LDL-C, Height, BMI, ALP, GGT, ALT, AST, ALB, GLB, DBIL, SBP, and DBP

*Abbreviations*: *CI* Confidence, *HR* Hazard ratios, *Ref* Reference

**Table 4:** The result of the two-piecewise linear regression model

	NAFLD (HR,95%CI)	*P*-value
Model I		
Fitting model by standard linear regression	1.21 (1.15, 1.28)	< 0.0001
Model II		
Fitting model by two-piecewise linear regression		
The inflection point of CAR	5.54	
≤ 5.54	2.20 (1.78, 2.73)	< 0.0001
> 5.54	1.10 (1.02, 1.18)	0.0159
*P* for the log-likelihood ratio test		< 0.001

Adjust for: Sex, Age, Cr, UA, TC, TG, HDL-C, LDL-C, Height, BMI, ALP, GGT, ALT, AST, ALB, GLB, DBIL, SBP, and DBP

*Abbreviations*: *CI* Confidence, *HR* Hazard ratios

### Subgroup analysis

This study then sought to better understand other possible influencing factors in the risk of FPG and NAFLD to identify potential special populations. First, the variables with *P* < 0.05 in univariate analysis were performed hierarchically (according to clinical significance or bisection). Second, although gender was not found to be statistically significant in univariate and multivariate analysis, according to previous experience, gender was a significant demographic variable for the incidence of NAFLD [2]. Therefore, gender was also included as a covariable for hierarchical analysis and interactive tests. The results are shown in Table 5. It can be observed that the interaction between FPG and height, TC, TG, LDL-C, HDL-C, ALP, GGT, ALT, AST, ALB, GLB was significant (*P* for interaction < 0.05). Of note, the effect of FPG on the incidence of NAFLD was more significant in people with short stature (HR:1.33, 95% CI:1.22–1.46, *P* < 0.0001, *P* for interaction = 0.0271).

**Table 5:** The effect size of FPG on NAFLD in prespecified and exploratory subgroups in each subgroup

Characteristic	No. of participants	HR (95%CI)	*P*-value	*P* for interaction
Age (years)				0.0757
≤ 30	2288	1.41 (1.24, 1.61)	< 0.0001	
> 30, ≤60	6179	1.20 (1.13, 1.28)	< 0.0001	
> 60	1300	1.16 (1.03, 1.32)	0.0184	
Sex				0.0633
Men	5022	1.17 (1.10, 1.25)	< 0.0001	
Women	4745	1.30 (1.19, 1.42)	< 0.0001	
BMI, kg/m^2^				0.5383
≤ 18.5	1115	2.51 (0.34, 18.43)	0.3668	
18.6–25	8652	1.23 (1.17, 1.29)	< 0.0001	
Height, m				0.0271
< 1.635	4807	1.33 (1.22, 1.46)	< 0.0001	
≥ 1.635	4960	1.18 (1.10, 1.25)	< 0.0001	
Cr, mmol/L				0.1052
< 104	9007	1.20 (1.14, 1.27)	< 0.0001	
≥ 104	760	1.43 (1.17, 1.75)	0.0005	
UA, μmol/L				0.5569
< 416	9352	1.21 (1.15, 1.27)	< 0.0001	
≥416	415	1.10 (0.80, 1.51)	0.5488	
TC, mmol/L				0.0003
< 4.39	4844	1.40 (1.29, 1.52)	< 0.0001	
4.39–5.2	4923	1.14 (1.07, 1.23)	0.0001	
TG, mmol/L				0.0203
< 0.93	4824	1.41 (1.26, 1.59)	< 0.0001	
0.93–1.7	4943	1.20 (1.13, 1.27)	< 0.0001	
LDL-C, mmol/L				0.0099
< 2.14	4883	1.35 (1.23, 1.47)	< 0.0001	
2.14–3.12	4884	1.16 (1.09, 1.24)	< 0.0001	
HDL-C, mmol/L				0.0024
≥ 1.04, < 1.48	4783	1.34 (1.24, 1.45)	< 0.0001	
≥ 1.48	4984	1.14 (1.05, 1.23)	< 0.0001	
ALP, U/L				0.0109
< 67	3531	1.50 (1.28, 1.75)	< 0.0001	
≥ 67	6236	1.20 (1.13, 1.27)	< 0.0001	
GGT, U/L				< 0.0001
< 40	9032	1.35 (1.26, 1.45)	< 0.0001	
≥ 40	735	1.07 (0.97, 1.18)	0.1677	
ALT, U/L				< 0.0001
< 40	9445	1.30 (1.22, 1.39)	< 0.0001	
≥ 40	322	1.01 (0.87, 1.16)	0.9230	
AST, U/L				< 0.0001
< 40	9579	1.31 (1.23, 1.39)	< 0.0001	
≥ 40	188	0.97 (0.81, 1.16)	0.7304	
ALB, g/L				0.0008
< 44.29	4269	1.16 (1.08, 1.24)	< 0.0001	
≥ 44.29	5498	1.40 (1.28, 1.53)	< 0.0001	
GLB, g/L				0.0031
< 29.44	4637	1.36 (1.25, 1.48)	< 0.0001	
≥ 29.44	5130	1.15 (1.08, 1.23)	< 0.0001	
DBIL, g/L				0.2803
< 2.1	2532	1.27 (1.15, 1.39)	< 0.0001	
≥ 2.1	7235	1.19 (1.12, 1.27)	< 0.0001	
SBP, mmHg				0.3633
< 140	8788	1.19 (1.12, 1.27)	< 0.0001	
≥ 140	979	1.26 (1.14, 1.40)	< 0.0001	
DBP, mmHg				0.2046
< 90	9253	1.19 (1.12, 1.26)	< 0.0001	
≥ 90	514	1.31 (1.15, 1.48)	< 0.0001	

Note 1: The above model adjusted for sex, Age, Cr, UA, TC, TG, HDL-C, LDL-C, Height, BMI, ALP, GGT, ALT, AST, ALB, GLB, DBIL, SBP, and DBP

Note 2: In each case, the model is not adjusted for the stratification variable

*Abbreviations*: *CI* Confidence, *HR* Hazard ratios

## Discussion

This study found that FPG was independently correlated with an increased risk of NAFLD (HR:1.32, 95% CI:1.28–1.37, *P* < 0.0001) in nonobese Chinese individuals with normal blood lipid levels. This association still exists after adjusting other covariates (HR:1.21, 95% CI:1.15–1.28, *P* < 0.0001), and it varies as with increasing FPG, the risk of NAFLD gradually increases. Several previous studies have reported similar results. In 2019, the Taiwanese scholar Hsu CL and his team found an association between FPG and NAFLD in 4000 nonobese people [18]. Additionally, a study from Sri Lanka reported the incidence of NAFLD in 34 local women; after post hoc analysis, it was found that there was a correlation between higher FPG and NAFLD [19]. Similar findings have been reported in China [24]; although these studies have highlighted that there is a positive correlation between FPG and NAFLD, these studies still have some limitations: (1) the adjustment of potential confounding factors, such as LDL-C, ALB, GLB, and DBP, is not sufficient; (2) the participants were not entirely nonobese, and the association between normal lipids and the incidence of NAFLD was not evaluated; (3) the nonlinear association among them has not been evaluated; and (4) these were not prospective studies, and it is difficult to guarantee the integrity and authenticity of the data, which could easily lead to more selection bias and information bias. Therefore, their conclusions were limited. The current study not only assessed the independent impact of FPG and NAFLD risk but also explored the nonlinear association between them. In the current literature, this was the first time that the nonlinear association between FPG and NAFLD has been explored, and the inflection point of FPG was calculated to be 5.54. When FPG ≤ 5.54, FPG was positively correlated with the risk of NAFLD (HR:2.20, 95% CI:1.78–2.73, *P* < 0.0001); similarly, when FPG > 5.54, the risk of NAFLD increased by 50% (HR:1.10, 95% CI:1.02–1.18, *P* = 0.0159) compared with the left side of the inflection point, and gradually leveled off (HR:1.10, 95% CI:1.02–1.18, *P* = 0.0159). Hierarchical analysis and interaction tests have helped us better understand other possible influencing factors in the risk of FPG and NAFLD. It can be observed that there is a significant interaction between FPG and height, TC, TG, LDL-C, HDL-C, ALP, GGT, ALT, AST, ALB, and GLB (*P* for interaction < 0.05). Furthermore, it is worth noting that in people with short stature, the positive correlation between FPG and NAFLD is stronger (HR:1.33, 95% CI:1.22–1.46, *P* < 0.0001, *P* for interaction = 0.0271). Studies have pointed out that adults with short stature are more likely to suffer from obesity, diabetes, and cardiovascular diseases, independent of BMI [25]. Additionally, a large prospective study in Finland (8746 people) found that short stature is associated with adverse changes in glucose metabolism [26]; this finding may be related to the existence of reduced beta-cell function and insulin sensitivity in people with short stature [26].

In a previous epidemiological survey of NAFLD in a nonobese Chinese population, the researchers analyzed 5562 nonobese participants without NAFLD. After a prospective follow-up for 5 years, the prevalence rate of NAFLD was 8.88%, which was similar to the prevalence of NAFLD in this survey (8.61%) [27]. However, in their study, no independent correlation was found between FPG and the occurrence of NAFLD; this outcome may be related to the fact that all of their participants were factory employees. Factory work requires more physical activity and reduces FPG levels (4.65 ± 0.39 VS 5.07 ± 0.70), thus reducing the risk of NAFLD. In addition, in a recent prospective study investigating the association of diabetes and blood glucose with chronic liver disease and liver cancer including 500,000 Chinese adult participants, diabetes and hyperglycemia were found to be associated with liver cancer and chronic liver disease [28]. This is critical information, whether relating to higher FPG, random plasma glucose or diabetes diagnosis, as this subgroup of patients with blood glucose metabolism disorder are more likely to suffer from NAFLD.

Several possible mechanisms may explain why higher blood glucose levels are linked to NAFLD in the future: (1) chronic hyperglycemia can induce liver toxicity by activating oxidative stress and endoplasmic reticulum stress responses leading to insulin resistance, steatosis and cellular demise [29]; (2) chronic hyperglycemia causes metabolic disorders in the liver, promotes mild inflammation, leads to insulin resistance (IR), and induces de novo fat synthesis in the liver [29]; and (3) IR may also lead to increased release of a variety of pro-inflammatory cytokines (such as interleukin-6 and leptin), thus increasing the risk of chronic liver disease [30].

The current study prospectively excluded people with BMI > 25, dyslipidemia and those diagnosed with NAFLD; it also followed participants for 5 years and found that height, BMI, TG, LDL-C, and FPG are strong independent risk factors for NAFLD in this particular population, and these factors may be related to fat distribution in Asians [10]. With the same BMI, Asians usually have a higher percentage of visceral fat and ectopic fat accumulation than people in Europe and North America [11]. More visceral fat and ectopic fat storage may lead to IR, which develops into NAFLD [31]. According to long-term clinical practice and literature study, some suggestions for the prevention of NAFLD may be effective. People with the following characteristics should pay more attention to monitoring biochemical blood indicators such as blood lipid and blood glucose and receive regular abdominal color doppler ultrasound examination: (1) BMI < 18.5 or high BMI within the normal range; (2) a high lipid level in the normal range; and (3) FPG > 5.27 mmol/L. Additionally, people with higher FPG need more aerobic exercise and dietary interventions, as they can increase muscle glucose transport activity and insulin stimulates muscle glycogen synthesis and reduces hepatic de novo lipogenesis and IR to reduce the risk of NAFLD [31, 32].

In the past, NAFLD was often regarded as a liver manifestation of metabolic syndrome. With the prevalence of obesity, the prevalence of metabolic syndrome and NAFLD has further increased [5]. However, some researchers have pointed out that NAFLD is the cause of the metabolic syndrome, due to the accumulation of large amounts of fat in the liver producing excess glucose and triglycerides and leading to metabolic disorders [33, 34]. In this study, the results seem to support that metabolic disorders precede the occurrence of NAFLD. Of course, this connection may also be two-way, and these conclusions need to be corroborated by further research.

### Study strengths and limitations

There are some notable strengths of this study. First, this was the first study to report the nonlinear association between FPG and the risk of NAFLD. Second, this study adopts a prospective design and makes strict statistical adjustments. At the same time, the original data and complete data were analyzed, which increases the reliability of the research conclusions. Third, this study included a relatively large sample size (nearly 10,000); the conclusion can be regarded as objective. Despite these strengths, the study has some potential weaknesses. First, FPG and other biochemical indicators were only measured during the physical examination, and dynamic changes in these levels over time were not considered. Second, insulin levels, which are closely associated with NAFLD, were not measured, so IR cannot be evaluated. Third, although ultrasonic screening is the most widely recommended method for its high sensitivity and specificity, it is still not accurate compared with liver biopsy and may underestimate the true incidence of NAFLD. Fourth, although a number of potential confounding factors had been adjusted, there were still some important factors that cannot be analyzed in this study due to the limitation of the original data, such as lifestyle, diet, and genetic factors. Fifth, as this research population was all Chinese, the external applicability of this study needs to be verified by more multicenter studies. Sixth, since nonalcoholic fatty liver (NAFL) and NASH could not be differentiated in this study, the results only apply to the assessment of NAFLD (in a broad sense) risk by FPG. Finally, because the single imputation method was used to deal with the missing data in this study, it may underestimate the degree of variation of the data and reduce the correlation between interpolation variables and other variables.

## Conclusion

In conclusion, FPG is an independent risk factor for NAFLD in a nonobese Chinese population with normal blood lipid levels. The findings of this study provide a convenient and useful marker for early prevention of NAFLD in nonobese people with normal blood lipids, which is helpful for the early detection of those with a high risk of NAFLD and provides early preventive measures.

## Supplementary information

**Additional file 1: Supplementary Table 1.** Collinearity diagnostics steps. **Supplementary Table 2.** Relationship between FPG and Ectopic NAFLD. **Supplementary Figure 1.** The nonlinear relationship between PFG and NAFLD for original data (A) and complete data (B). Original data adjust for: Sex, Age, ALP, GGT, ALT, AST, ALB, GLB, DBIL, CR, UA, TG, HDL-C, LDL-C, Height, BMI, SBP, and DBP. Complete data adjust for: Sex, Age, Cr, UA, TC, TG, HDL-c, LDL-c, Height, BMI, ALP, GGT, ALT, AST, ALB, GLB, DBIL, SBP, and DBP. **Supplementary Table 3.** The result of the two-piecewise linear regression model.

## Data Availability

Data can be downloaded from the ‘DATADRYAD’ database (www.Datadryad.org, 10.5061/dryad.1n6c4).

## Abbreviations

FPG: Fasting plasma glucose
NAFLD: Nonalcoholic fatty liver disease
NASH: Nonalcoholic steatohepatitis
BMI: Body mass index
SBP: Systolic blood pressure
DBP: Diastolic blood pressure
BUN: Blood urea nitrogen
Cr: Creatinine
UA: Uric acid
TC: Total cholesterol
TG: Triglyceride
HDL-C: High-density lipoprotein cholesterol
LDL-C: Low-density lipoprotein cholesterol
ALP: Alkaline phosphatase
GGT: Gamma-glutamyl transferase
ALT: Alanine aminotransferase
AST: Aspartate aminotransferase
TP: Total Protein
ALB: Albumin
GLB: Globulin
TB: Total bilirubin
DBIL: Direct bilirubin
VIF: Variance inflation factor
HR: Hazard ratios
CI: Confidence interval
IR: Insulin resistance
STROBE: Strengthening the Reporting of Observational Studies in Epidemiology
