# Supplementary material for: Association between fasting plasma glucose and nonalcoholic fatty liver disease in a nonobese Chinese population with normal blood lipid levels: a prospective cohort study
Source: Lipids Health Dis. 2020 Jun 20;19:145. doi: 10.1186/s12944-020-01326-3 (PMC7306139; doi:10.1186/s12944-020-01326-3)
Supplement: Supplementary file 1 — Additional file 1: Supplementary Table 1. Collinearity diagnostics steps. Supplementary Table 2. Relationship between FPG and Ectopic NAFLD. Supplementary Figure 1. The nonlinear relationship between PFG and NAFLD for original data (A) and complete data (B). Original data adjust for: Sex, Age, ALP, GGT, ALT, AST, ALB, GLB, DBIL, CR, UA, TG, HDL-C, LDL-C, Height, BMI, SBP, and DBP. Complete data adjust for: Sex, Age, Cr, UA, TC, TG, HDL-c, LDL-c, Height, BMI, ALP, GGT, ALT, AST, ALB, GLB, DBIL, SBP, and DBP. Supplementary Table 3. The result of the two-piecewise linear regression model. [file 12944_2020_1326_MOESM1_ESM.docx]

Supplementary Table 1. Collinearity diagnostics steps.

| Variables | Step1 | Step2 | Step3 |
| --- | --- | --- | --- |
| Sex | 1.1 | 1.1 | 1.1 |
| Age | 1.1 | 1.1 | 1.1 |
| BUN | 1.4 | 1.4 | 1.4 |
| Cr | 1.6 | 1.6 | 1.6 |
| UA | 1.6 | 1.6 | 1.6 |
| FPG | 1.2 | 1.2 | 1.2 |
| TC | 4.7 | 4.7 | 4.7 |
| TG | 1.4 | 1.4 | 1.4 |
| HDL-C | 2.1 | 2 | 2 |
| LDL-C | 4.5 | 4.5 | 4.5 |
| Weight | 231.9 | 231.9 | NA |
| Height | 97.5 | 97.5 | 1.4 |
| BMI | 105 | 105 | 1.3 |
| ALP | 1.2 | 1.2 | 1.2 |
| GGT | 1.3 | 1.3 | 1.3 |
| ALT | 3.1 | 3.1 | 3.1 |
| AST | 3.2 | 3.2 | 3.2 |
| TP | 1969417.8 | NA | NA |
| ALB | 882267.5 | 1.2 | 1.2 |
| GLB | 1757409.8 | 1.1 | 1.1 |
| TB | 1.8 | 1.8 | 1.8 |
| DBIL | 1.9 | 1.9 | 1.9 |
| SBP | 2.5 | 2.5 | 2.5 |
| DBP | 2.2 | 2.2 | 2.2 |

abbreviations as in Table 1

Supplementary Table 2. Relationship between FPG and Ectopic NAFLD.

| Variable |  | Original data |  | Complete data |
| --- | --- | --- | --- | --- |
|  |  | HR (95%CI) *P*-value |  | HR (95%CI) *P*-value |
| FPG |  | 1.29 (1.19, 1.40) <0.0001 |  | 1.21 (1.15, 1.28) <0.0001 |
| FPG(quartile) |  |  |  |  |
| Q1 |  | Ref |  | Ref |
| Q2 |  | 1.42 (0.95, 2.11) 0.0834 |  | 1.29 (1.01, 1.66) 0.0450 |
| Q3 |  | 1.26 (0.85, 1.85) 0.2468 |  | 1.41 (1.11, 1.79) 0.0054 |
| Q4 |  | 2.15 (1.50, 3.09) <0.0001 |  | 2.25 (1.79, 2.82) <0.0001 |
| *P* for trend |  | <0.0001 |  | <0.0001 |

Original data adjust for: Sex, Age, ALP, GGT, ALT, AST, ALB, GLB, DBIL, CR, UA, TG, HDL-C, LDL-C, Height, BMI, SBP, and DBP.

Complete data adjust for: Sex, Age, Cr, UA, TC, TG, HDL-c, LDL-c, Height, BMI, ALP, GGT, ALT, AST, ALB, GLB, DBIL, SBP, and DBP.

Abbreviations: CI: confidence; HR: hazard ratios; Ref: reference.


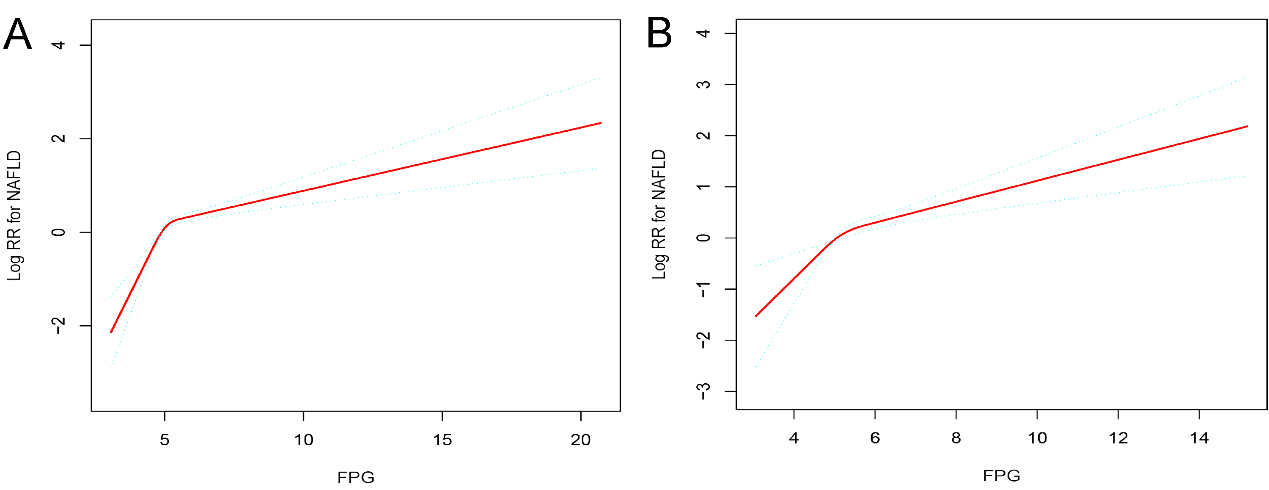


Supplementary Figure 1: The nonlinear relationship between PFG and NAFLD for original data (A) and complete data (B). Original data adjust for: Sex, Age, ALP, GGT, ALT, AST, ALB, GLB, DBIL, CR, UA, TG, HDL-C, LDL-C, Height, BMI, SBP, and DBP. Complete data adjust for: Sex, Age, Cr, UA, TC, TG, HDL-c, LDL-c, Height, BMI, ALP, GGT, ALT, AST, ALB, GLB, DBIL, SBP, and DBP.

| Supplementary Table 3. The result of the two-piecewise linear regression model | | | |  |  | |
| --- | --- | --- | --- | --- | --- | --- |
|  | Complete data |  | Original data | | | |
|  | NAFLD (HR,95%CI ) | *P*-value | NAFLD (HR,95%CI ) | | | *P*-value |
| Model I | | |  | | |  |
| Fitting model by standard linear regression | 1.21 (1.15, 1.28) | <0.0001 | 1.29 (1.19, 1.40) | | | <0.0001 |
| Model II |  |  |  | | |  |
| Fitting model by two-piecewise linear regression | | |  | | |  |
| The inflection point of CAR | 5.54 |  | 5.51 | | |  |
| ≤5.54 | 2.20 (1.78, 2.73) | <0.0001 | 1.94 (1.39, 2.69) | | | <0.0001 |
| >5.54 | 1.10 (1.02, 1.18) | 0.0159 | 1.20 (1.07, 1.34) | | | 0.0015 |
| *P* for the log-likelihood ratio test |  | <0.001 |  | | | 0.01 |

Original data adjust for: Sex, Age, ALP, GGT, ALT, AST, ALB, GLB, DBIL, CR, UA, TG, HDL-C, LDL-C, Height, BMI, SBP, and DBP.

Complete data adjust for: Sex, Age, Cr, UA, TC, TG, HDL-c, LDL-c, Height, BMI, ALP, GGT, ALT, AST, ALB, GLB, DBIL, SBP, and DBP.

Abbreviations: CI: confidence; HR: hazard ratios;
